# Metagenomic Evidence for a *Methylocystis* Species Capable of Bioremediation of Diverse Heavy Metals

**DOI:** 10.3389/fmicb.2018.03297

**Published:** 2019-01-09

**Authors:** Ling-Dong Shi, Yu-Shi Chen, Jia-Jie Du, Yi-Qing Hu, James P. Shapleigh, He-Ping Zhao

**Affiliations:** ^1^College of Environmental and Resource Sciences, Zhejiang University, Hangzhou, China; ^2^Zhejiang Province Key Laboratory for Water Pollution Control and Environment, Zhejiang University, Hangzhou, China; ^3^MOE Key Lab of Environmental Remediation and Ecosystem Health, College of Environmental and Resource Sciences, Zhejiang University, Hangzhou, China; ^4^Department of Microbiology, Cornell University, Ithaca, NY, United States

**Keywords:** heavy metals, reductase, methanotroph, metagenomics, *Methylocystis*

## Abstract

Heavy metal pollution has become an increasingly serious problem worldwide. Co-contamination with toxic mercury (Hg) and arsenic (As) presents a particularly difficult bioremediation trouble. By oxidizing the greenhouse gas methane, methanotrophs have been demonstrated to have high denitrification activity in eutrophic waters, indicating their possible potential for use in bioremediation of Hg(II) and As(V) in polluted water. Using metagenomics, a novel *Methylocystis* species (HL18), which was one of the most prevalent bacteria (9.9% of the relative abundance) in a CH_4_-based bio-reactor, is described here. The metagenomic-assembled genome (MAG) HL18 had gene products whose average amino acid identity against other known *Methylocystis* species varied from 69 to 85%, higher than the genus threshold but lower than the species boundary. Genomic analysis indicated that HL18 possessed all the genes necessary for the reduction of Hg(II) and As(V). Phylogenetic investigation of mercuric reductase (MerA) found that the HL18 protein was most closely affiliated with proteins from two Hg(II)-reducing bacteria, *Bradyrhizobium* sp. strain CCH5-F6 and *Paracoccus halophilus*. The genomic organization and phylogeny of the genes in the As(V)-reducing operon (*arsRCCB*) had significant identity with those from a As(V)-reducing bacterium belonging to the *Rhodopseudomonas* genus, indicating their reduction capability of As(V). Further analysis found that at least eight genera of methanotrophs possess both Hg(II) and As(V) reductases, illustrating the generally overlooked metabolic potential of methanotrophs. These results suggest that methanotrophs have greater bioremediation potential in heavy metal contaminated water than has been appreciated and could play an important role in the mitigation of heavy metal toxicity of contaminated wastewater.

## Introduction

Mercury (Hg), a toxic and bio-accumulative trace metal, has increased remarkably because of anthropogenic activities such as mining and fossil fuel combustion, and as a result seriously threatens the environment and is a growing public health concern (Streets et al., 2011). Inorganic mercury ion (Hg(II)), with high water solubility, can irritate the gut, cause kidney damage, and will bind to thiol group of essential proteins and consequently inactivate them (Langford and Ferner, 1999; Mathema et al., 2011). Industrial activities can also produce arsenic (As), a cancerogenic and mutagenic element, resulting in a co-contaminated waste stream and further polluting natural waters (Rahman et al., 2014). For example, Hg in shallow-water lagoons have concentrations of 0.38–231 ng/L, and As concentration in natural waters, including freshwater and groundwater, ranges from 0.50 to 5000 μg/L (Smedley and Kinniburgh, 2002; Faganeli et al., 2012). This co-occurrence makes the treatment of contaminated waters much more challenging.

The most common forms of Hg in waters are divalent Hg(OH)_2_ and HgCl_2_, while the inorganic arsenate (As(V)) and arsenite (As(III)) are the primary types of As in aquatic environments (Lievremont et al., 2009). Considering that (1) the higher valency forms of Hg and As are more soluble and bio-available; (2) Hg(0) vapor can simply volatilize from waters (Munthe and Mcelroy, 1992), and that As(III) can easily precipitate with sulfide or sulfide-containing materials and be removed by centrifugation or filtration (Newman et al., 1997), it is the bio-reduction of Hg(II) and As(V) that is the major focus of Hg/As remediation efforts.

Many Gram-positive and Gram-negative bacteria are able to reduce Hg(II) with mercuric reductase. The operons whose gene products confer mercury-resistance contain several functional genes such as *merA, merB, merP, merT, merD, merR, merC* et al., depending on the particular strain (Dash and Das, 2012). Typically, MerR and MerD initially detect Hg(II) and regulate the transcription of other genes (Huang et al., 2002; Champier et al., 2004). Then, MerP, MerT, and MerC transport the mercury from the extracellular space into the cytoplasm (Liebert et al., 1997; Powlowski and Sahlman, 1999; Rossy et al., 2004). Finally, MerA and MerB detoxify inorganic and organic mercury compounds by reducing the ionic form thus generating volatile Hg^0^ which is expelled from cells (Osborn et al., 1997).

As(V) reduction involves two metabolic systems: the *arr* respiration system or the *ars* detoxification system (Mukhopadhyay and Rosen, 2002). Saltikov and Newman (2003) reported that the respiratory As(V)-reducing ability of *Shewanella* sp. strain ANA-3 was carried out by a two-subunit enzyme. The smaller subunit of the dimer, ArrB, contains four 4Fe–4S clusters and transfers electrons through the respiratory chain to the larger catalytic subunit ArrA, which utilizes a molybdenum cofactor and then reduces As(V) (Silver and Phung, 2005). The *ars* system is cytoplasmic and thus not coupled to respiration. This operon has three core genes (*arsRBC*), encoding a transcriptional repressor, a transmembrane efflux pump, and an As(V) reductase, respectively (Lievremont et al., 2009). A larger version composed of five genes (*arsRDABC*) has also been reported (Martin et al., 2001). ArsA is an ATPase which provides energy to the pump for discharge of As(III) while ArsD is an arsenic chaperone which transfers the As(III) to ArsA and activates the ArsAB As(III) excretion pump (Lin et al., 2007).

Microbes with these metal reduction pathways have been utilized to remediate Hg/As contaminated waters. For example, Yu et al. (2014) reported *Staphylococcus epidermidis* strain Lzu-02 could reduce Hg(II) to less toxic volatile Hg(0) using glucose as electron donor. Bachate et al. (2009) isolated twenty bacteria which contained the *arsC* gene and were capable of reducing As(V) to As(III) with gluconate as the electron donor. More recently, some studies have reported that methanotrophs possess a Hg(II)-reducing system. Boden and Murrell (2011) demonstrated that *Methylococcus capsulatus* (Bath) was able to oxidize CH_4_ in the presence of Hg(II) although neither the production of Hg(0) nor the involvement of MerA was confirmed. *Methylosinus trichosporium* OB3b and *Methylocystis* strain SB2 were reported to have the capability to synthesize methanobactin to detoxify Hg through complexation (Vorobev et al., 2013; Baral et al., 2014). However, whether methanotrophs have the ability to reduce heavy metals remains to be directly demonstrated.

In the present study, we identified a novel bacterium belonging to *Methylocystis* genus from a CH_4_-fed bioreactor by metagenomic analysis, and mainly focused on its metals reduction pathways instead of generally basic characterizations such as morphology. Analysis of MAG found it has both Hg- and As-reduction gene operons, suggesting it will be of practical use for heavy metal bioremediation in contaminated waters.

## Materials and Methods

### Sample Enrichment and DNA Isolation

This microbial community was enriched in a CH_4_-fed membrane biofilm bio-reactor using the original culture from Lai et al. (2016a). Biofilm samples were collected through cutting off several ∼10-cm sections from fibers (Zhong et al., 2017). We then placed the sections into a centrifuge tube with DNA-free water, and vortexed at the highest speed for 15 min to detach the biofilm from the fibers as described by Lai et al. (2016c). DNA from the biofilm was isolated using the PowerSoil DNA Isolation Kit (MO BIO) following the manufacturer’s instructions (Lai et al., 2016b).

### Metagenome Sequencing and Genome Reconstruction

Shotgun metagenomic sequencing (2 × 150 bp) was performed using the Illumina HiSeq X Ten. The Illumina TruSeq DNA Nano Library Prep Kit was used for library preparation. About 205 million paired-end reads were generated. The quality of the raw data was confirmed using FastQC^1^. We trimmed the partial primers and adapters from the reads using the BBmap package^2^, and further removed the reads which: (i) contained the spike-in PhiX sequences; (ii) were low-quality (Phred Quality Score < 15) or short (<30 bp); (iii) had low complexity. All reads that passed quality control were assembled into contigs using metaSPAdes (Bankevich et al., 2012), yielding one assembly. Quality-controlled reads were mapped to assembled contigs (>500 bp) through BBmap and contigs were annotated using an in-house pipeline, meta-annotator. Metabat^3^ and CheckM^4^ were used to bin the contigs and check the completeness of bins, respectively.

### Phylogenetic Analysis

Phylogenetic trees of relevant proteins were constructed using MEGA 6 with a multi-step approach (Tamura et al., 2013; Chen et al., 2016). Briefly, functional amino acid sequences were initially aligned, and then fragmentary and non-overlapping regions were removed. Phylogenetic trees were subsequently generated using the neighbor-joining algorithm based on distance analysis by MEGA 6 program packages. Reference proteins were found in the NCBI database through BLASTp algorithm. The accuracy of inferred topologies was tested by bootstrap re-sampling of 1000 replicates using the same distance model.

### Gene Abundance Analysis

Genes for arsenate reductase (*arsC*) and the house-keeping RNA polymerase β subunit (*rpoB*) were searched in the six-frame translated metagenomic contigs in HL18 genome, using hmmsearch with e-5 of the cutoff *e*-value. The ArsC (PF03960, PF00462) and the RpoB (TIGR02013, TIGR03670) hidden Markov models were downloaded from the Pfam and TIGRFAMs database, respectively (Haft et al., 2013; Finn et al., 2016). The metagenomic reads after quality control were mapped against the *arsC* and *rpoB* nucleotide gene sequences using bowtie2 (Langmead and Salzberg, 2012). Picard^5^was used to remove PCR duplicates in the mapping files, and then BEDTools was applied to calculate coverage values for the extracted genes (Quinlan and Hall, 2010). To avoid that longer genes had higher values due to their length, the coverages were normalized to the length of corresponding genes, and further normalized to the total number of the *rpoB* gene, yielding an approximate number of each extracted *arsC* gene in HL18 assembled genome.

### Data Availability

All raw Illumina sequences were submitted to the Sequence Read Archive (SRA) under Accession No. SRP136696 as described by Ontiveros-Valencia et al. (2014).

## Results

### Genome Reconstruction and Phylogeny

**FIGURE 1:**
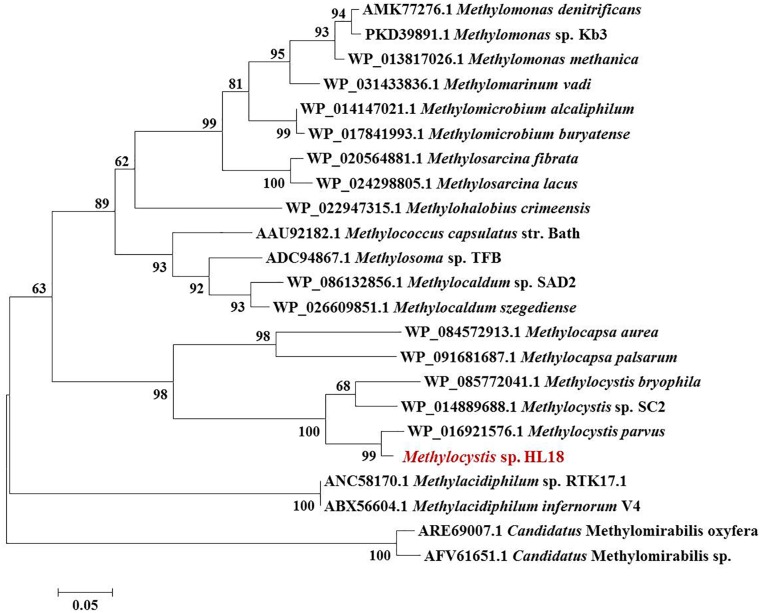
Phylogenetic tree of PmoA based on amino acid sequences. The microorganism in red font was the one recovered in this study. PmoA of *Methylomirabilis* genus belonging to NC10 phylum were used as the outgroup members of this tree. Numbers indicated the bootstrap support (1000 replicates).

**FIGURE 2:**
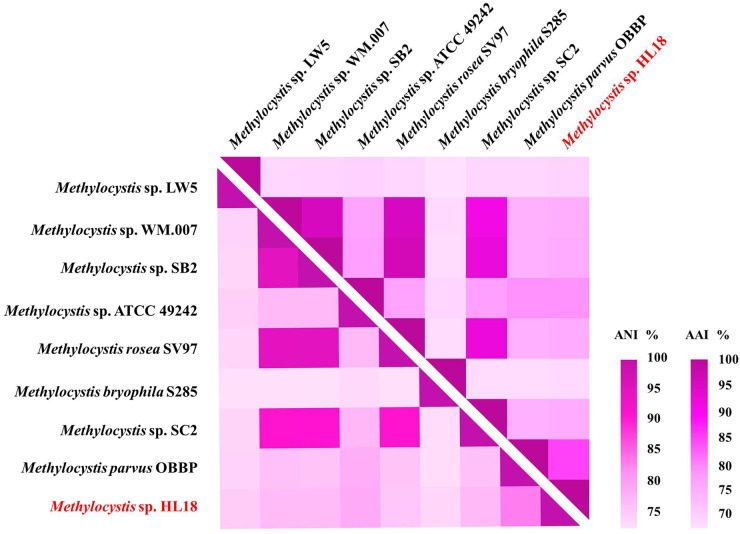
Heatmap of genome relatedness between HL18 and other methanotrophs belonging to *Methylocystis*, generated by HemI (Deng et al., 2014). The average nucleotide identity (ANI; lower triangle) and average amino acid identity (AAI; upper triangle) between pairs of genomes were calculated using JSpeciesWS (Richter et al., 2016) and CompareM package (https://github.com/dparks1134/CompareM), respectively.

Metagenome sequencing, assembly, and binning reconstructed a number of high quality draft genomes from the CH_4_-fed bioreactor. The most abundant microorganism responsible for methane oxidation was a member of the *Methylocystis*, a Type II methanotroph, which accounted for 9.9% of the community (by calculating the sequence percentage). This MAG, referred to as HL18, contained 69 contigs, with an N50 of 97.898 Kbp. The total genome size was 3.816 Mbp (Table 1). HL18 was estimated to have >99% completeness and <1% contamination by CheckM, indicating a nearly complete genome. Unfortunately, the 16S rRNA gene was not present in the assembly. Skennerton et al. (2015) reported the consistent phylogenetic placement using *pmoA* gene to replace the *16S rDNA*. Thus we adopted the *pmoA* gene encoding protein as the criterion to analyze the phylogeny of HL18. Figure 1 shows the phylogenetic tree of the PmoA based on amino acid sequences, with organisms from the candidate division NC10 as the outgroup members. HL18 was located within other members of the *Methylocystis* genus, and most closely affiliated with *Methylocystis parvus*. However, the average nucleotide identity (ANI) between HL18 and *Methylocystis parvus* was about 83%, which was only slightly above that (74–80%) of other members of this genus (Figure 2). ANI values for species circumscription are typically 95–96% (Richter and Rosselló-Móra, 2009), while between different species within the same genus these values range from 62 to 100% (Kim et al., 2014). Additionally, the average amino acid identity (AAI) of HL18 and other species varied from 69 to 85% (Figure 2), higher than the genus threshold but lower than the species boundary (Konstantinidis and Tiedje, 2007). These results indicated HL18 should be a novel species belonging to *Methylocystis* genus.

**Table 1:** Overview of the HL18 assembled genome.

Draft genome information	
Genome size	3.816 Mbp
Number of contigs	69
N50 (contigs)	97.898 Kbp
GC content	64.89%
completeness	99.37%
Contamination	0.95%


### Potential of Inorganic Mercury Reduction

**FIGURE 3:**
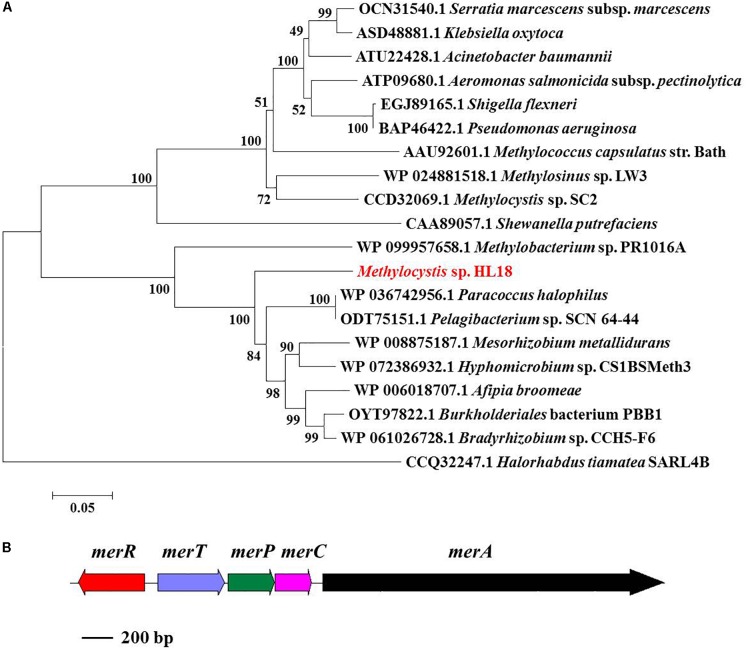
**(A)** Phylogenetic tree of MerA based on amino acid sequences and **(B)** *mer* operon containing five genes (*merR*, *merT*, *merP*, *merC*, and *merA*). MerA of archaea *Halorhabdus tiamatea* was used as the outgroup member of this tree. Numbers indicated the bootstrap support (1000 replicates).

*merA*, the gene encoding the inorganic Hg(II) reductase, was found in the HL18 MAG. Phylogenetic analysis (Figure 3A) demonstrated that MerA has the same ancestor as similar proteins in *Bradyrhizobium* sp. strain CCH5-F6 and *Paracoccus halophilus*. Moreover, *merR*, *merT*, *merP*, and *merC* (Figure 3B) were found upstream of *merA*, whose products might contribute to the complete metabolic pathway for Hg(II) reduction. We compared GC content of the mercury-resistant operon, the contig where operon located, and the whole genome (Supplementary Figure S1), to test the possible sources of other microbes resulting from mis-assembly or mis-binning, even though the estimated contamination value was very low (0.95%). The differences between these contents were small (0.01–1.21%), suggesting this region had not been incorrectly assigned to HL18.

### Potential of Arsenate Reduction

**FIGURE 4:**
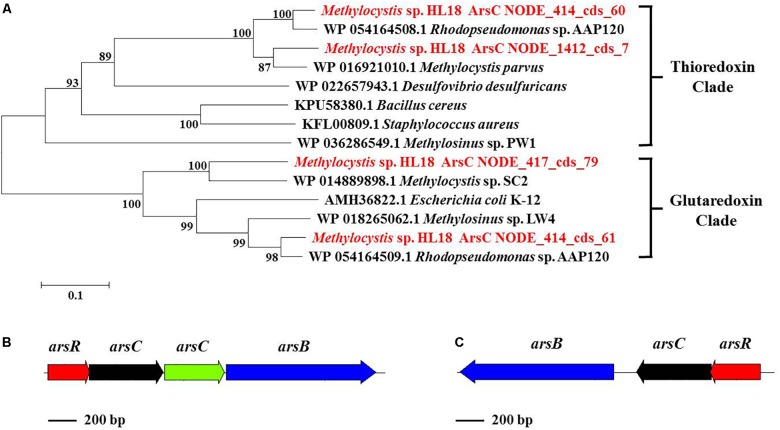
**(A)** Phylogenetic tree of ArsC based on amino acid sequences, **(B)** *ars* operon containing four genes (*arsR*, *arsC*, *arsC*, and *arsB*) and **(C)** another *ars* operon containing three genes (*arsR*, *arsC*, and *arsB*). ArsC sequences recovered in this study from the metagenome were in red font and suffixed with their contig names. Numbers indicated the bootstrap support (1000 replicates).

In addition to Hg(II) reductase, HL18 also possessed the As(V) reductase (ArsC) (Figure 4). There were four *arsC* genes distributed on three contigs (Figure 4A). Two reductases belonged to the glutaredoxin (Grx) clade, whose prototype is the enzyme of *Escherichia coli* plasmid R773 (Gladysheva et al., 1994; Martin et al., 2001); the other two were associated with the thioredoxin (Trx) clade having ArsC of *Staphylococcus aureus* plasmid pI258 as prototype (Ji et al., 1994). We compared the GC content of the *arsC* operons, corresponding contigs, and the whole genome, to preliminarily determine whether the affiliation was rational. Supplementary Figure S1 reveals that one *arsC* gene, labeled as NODE_417_cds_79, has quite different GC content with those of both the relevant contig and the whole genome. This deviation (∼9%) implied the possible contamination of this *arsC* gene. Besides, no other associated genes (e.g., *arsB*, the gene encoding the As(III) transporter) could be found nearby this gene, thus it was discarded in the subsequent analysis. For the remaining three genes, two *arsC* were adjacent to one another on the same contig, with each having closest affiliations to the proteins in *Rhodopseudomonas* sp. AAP120, while the other was on a separate contig and its closest affiliation was *Methylocystis parvus*. Genes encoding the regulator protein ArsR and the As(III) transporter ArsB could also be found in the vicinity of *arsC* genes (Figures 4B,C), forming two operons encoding proteins whose products are involved in As(V) reduction.

Furthermore, the *arsC* gene number was calculated based on the single-copy gene *rpoB*, the house-keeping gene for the RNA polymerase β subunit. Average abundances of *arsC* ranged from 3.6 to 3.9 fold more than the number of *rpoB*, leading to total ∼11 fold higher in HL18 MAG. The effect of the *ars* operon on As(V) resistance has been reported to be cumulative (Diorio et al., 1995; Cai et al., 1998). Two operons responsible for As(V) reduction and high abundances of *arsC* are consistent with the increased As(V)-resistance of HL18, suggesting it may have better efficiency to bio-remediate As(V) contaminated wastewater.

### Potential for Metal Resistance in Other Methanotrophs

The presence of Hg(II) and As(V) reductases in other methanotrophs has not been reported. Analysis of available genomes found that members of at least eight genera that had the capacity to oxidize methane possessed both Hg(II) and As(V) reductases. Identities of relevant reductases between HL18 and 10 type species were calculated using BLASTp. Primary sequence identity of the Hg(II) reductases ranged from 39–97% while As(V) reductases were 33–97% similar (Supplementary Figures S2–S4). Phylogenetic analysis demonstrated significant diversity of both reductases within methanotrophs as shown by the fact that many reductase clades occur (Supplementary Figures S5–S7). The MerA of HL18, along with that of *Methylosarcina lacus*, did not form clades with any of the other proteins, revealing the divergent sequences of these two reductases. Similarly, a variety of methanotrophic genera belonging to seven taxonomic families contained different clades of As(V) reductases. Two thioredoxin-dependent enzymes from HL18 were found clustered with proteins from *Methylocystaceae* and *Beijerinkiaceae* clusters (Supplementary Figure S6). This result suggests that the gene encoding ArsC (NODE_414_cds_60) was derived from horizontal gene transfer rather than the duplication of another ArsC (NODE_1412_cds_7) gene. Interestingly, the glutaredoxin-clade ArsC (NODE_414_cds_61), which is directly adjacent to NODE_414_cds_60 also clustered with enzymes in the *Beijerinkiaceae* cluster, lending further support to the possibility that these genes were acquired by lateral gene transfer (Supplementary Figure S7).

## Discussion

**FIGURE 5:**
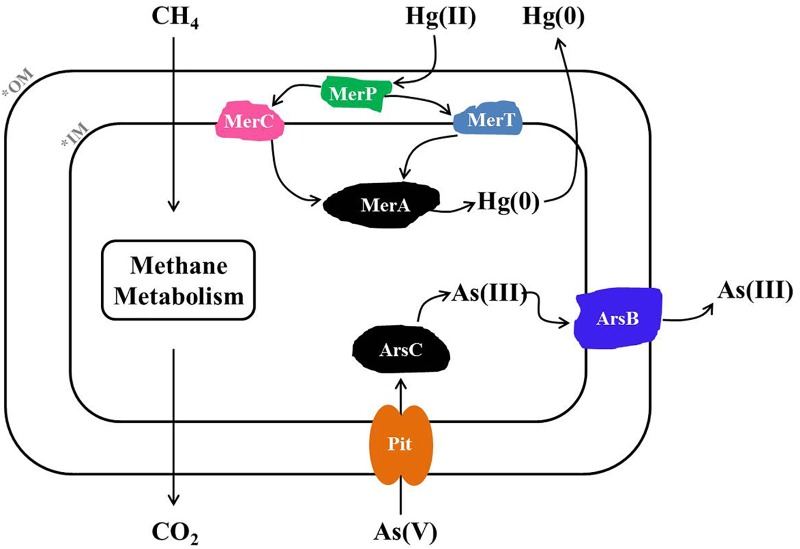
Metabolic pathway of Hg(II) and As(V) reduction inferred from the HL18 genome sequence. OM, outer membrane; IM, inner membrane; MerA, mercuric reductase; MerT, mercuric transport protein MerT; MerC, mercuric transport protein MerC; MerP, mercuric transport protein periplasmic component; Pit, phosphate transporter; ArsC, arsenate reductase; ArsB, arsenic efflux pump protein.

Based on metagenome sequencing, we generated a MAG from a methanotroph (HL18) found in a CH_4_-fed bioreactor that is a new member of the genus *Methylocystis*. HL18 was the highest occurring methanotroph in the community and contained both Hg(II) and As(V) reductases, revealing a multiplicity of heavy metal reduction capacities. The assembled genome of HL18 contained the genes necessary for detoxication of Hg(II) including the mercuric reductase MerA. The MerA in HL18 shares significant primary sequence identity (80%) with that from *Bradyrhizobium* sp. strain CCH5-F6 which has been reported capable of Hg(II) reduction, implying that HL18 also had the potential to reduce Hg(II) (Wang et al., 2013). Moreover, the protein with the high sequence identity (77%) to the MerA in HL18 is found in *Paracoccus halophilus*, a bacterium able to tolerate Hg(II), also providing evidence for its Hg(II)-reducing potential (Lapanje et al., 2010). Based on previous work and analysis of the HL18 MAG, when HL18 is exposed to mercury, MerR would detect the presence of Hg(II) and activate the transcription of downstream genes (Huang et al., 2002). This would enable the transport of Hg(II) into the cytoplasm via MerP where it would be subsequently transferred to the inner membrane bound protein MerT and MerC (Hamlett et al., 1992). Hg(II) would then be transferred to MerA and further reduced to elemental Hg(0) by an NAD(P)H-dependent reaction (Dash and Das, 2012). The proposed metabolic pathway based on the genes in HL18 is shown in Figure 5.

Likewise, HL18 also contains As(V) reduction potential conferred by two distinct As(V)-reducing operons, *arsRCCB* and *arsRCB*. The difference of *arsC* number in two operons is not unreasonable since a variety of arrangements of As(V)-resistance genes have been discovered in other microorganisms (Martin et al., 2001; Li and Krumholz, 2007; Lievremont et al., 2009). The structure and phylogeny of *arsRCCB* operon in HL18 were very similar with those in *Rhodopseudomonas* sp. AAP120 (BioProject Accession No. PRJNA255569). While the arsenate reductase activity of *R.* sp. AAP120 has not been reported, its relative *R. palustris* C1 was found to reduce As(V), with a specific activity of 12.07 U mg^-1^ protein, which was comparable to that of the As(V)-reducing bacterium *Pseudomonas* sp. (12.15 U mg^-1^ protein) (Srivastava et al., 2010; Nookongbut et al., 2017). Based on the similarity of the proteins in HL18 to proteins with known As(V)-reducing activity, it is very likely that these proteins in HL18 are active as well. The gene organization of the other *ars* operon in HL18 was very similar other than the absence of one copy of *arsC*. The closest affiliation of the *arsC* in this operon was with a protein from *Methylocystis parvus*. To date, there have been no articles reporting As(V) reducing capacity in methanotrophs. However, all genes required for As(V) reduction could be found flanking the *arsC* in both operons (Figures 4B,C). Furthermore, we inoculated the consortium which contained HL18 (Supplementary Table S1) in serum bottles along with CH_4_ and As(V), and observed significant reduction of As(V) that did not happen without CH_4_ in presence or with killed inoculum (Supplementary Figure S8). Among the consortium, the highest abundant bacterium was related to genus *Pseudoxanthomonas*, of which the closest member was *P. suwonensis* with the ANI of 76.7%. Although an isolate from this genus has been reported able to reduce As(V), it was phylogenetically distant from *P. suwonensis* (Mohapatra et al., 2018), implying the different metabolisms between species in the same genus. Additionally, *arsB* whose gene product could expel As(III) and thus confer As-resistance (Fu et al., 2009), was not found in the assembled genome of putative *Pseudoxanthomonas* sp., indicating this species had no capacibility for As(V) reduction in our experiment. Likewise, *arsC*, *arsR*, *arsB* in the genomes of putative *Meiothermus* sp. distributed dispersely and did not constitute an operon, excluding its possibility for As(V) reduction. Inversely, other four bacteria, including HL18, possessed different forms of As(V)-reducing operons, implying their active role in CH_4_-based As(V) reduction. While it is possible that one or several alternate genera were the primary As(V)-reducing strains, we could not exclude the circumstance that *Methylocystis* sp. HL18 may have been responsible for some of the As(V) reduction. Based on the available genes, we propose that when As(V) is present, inactivation of *arsR* will result in the expression of the *ars* system (Rosenstein et al., 1992). As(V) would then be transported from outside to the cytoplasm through the phosphate transporter Pit and then reduced by ArsC (Willsky and Malamy, 1980; Martin et al., 2001). The product As(III) was then be transported out of the cytoplasm via ArsB (Rosen, 2002).

In addition to HL18, multiple methanotrophic bacteria also contain Hg(II) and As(V) reductases. However, the Hg(II) reductase of HL18 was notably different with proteins of other *Methylocystis* species, only sharing ∼47% identity (Supplementary Figure S2). These identities were much smaller than those (>80%) between other species within the same genus (Supplementary Figure S2), and that (80%) with MerA from *Bradyrhizobium* sp. CCH5-F6. Lateral gene transfer may explain why the MerA from HL18 is more related to the *Bradyrhizobium* protein instead of proteins from other *Methylocystis*. Unlike the MerA, As(V) reductases from the same genus, *Methylocystis*, have the highest sequence relatedness with the ArsC from HL18 (Supplementary Figures S3, S4). These arsenate reductase genes are widespread in methanotrophic bacteria, with at least seven families and more than 10 genera (Supplementary Figures S6, S7). This distribution implies the common occurrence of As(V) reduction in methanotrophs.

Very recently, Ettwig et al. (2016) and Cai et al. (2018) described that archaea related to *Candidatus* Methanoperedens nitroreducens were able to couple the reduction of iron/manganese ions to the oxidation of methane, demonstrating additional possibilities of high valent heavy metals reduction of methanotrophs. Although there is yet no direct biochemical evidence illustrating Hg(II) and As(V) reduction of methanotrophs, genome information strongly supports the conclusion that a variety members of this group have this physiological capacity. In addition, some members of *Methylomonas* genus contain both metals reductases as well as denitrification enzymes, and one species in this genus (*Methylomonas denitrificans* sp. strain FJG1) has been reported to reduce nitrate (Kits et al., 2015), though the capacity for Hg(II) or As(V) reduction across members of the genus are unknown. Meanwhile, pure culture of *Methylococcus capsulatus* (Bath) has been demonstrated possessing the mercuric reduction potential (Boden and Murrell, 2011). Our supplemented experimental result preliminarily confirmed arsenate reduction ability of the consortium which contains high abundance of HL18. These results together illustrate that many methanotrophs, including HL18, have an unappreciated metabolic potential and versatile capabilities for bio-remediation. Furthermore, Hg and As are globally dispersed (Wedepohl, 1995; Mukhopadhyay et al., 2002) and very likely co-occur with methane, indicating that the links between the carbon, arsenic, and mercury cycles may be more common than previously recognized. However, further ecophysiological experiments are required to confirm the function of these methanotrophs both in lab reactors and natural ecology.

## Author Contributions

L-DS and H-PZ constructed the experiments, drafted, and revised the manuscript. JS helped to analyze the data and revise the manuscript. Y-SC, J-JD, and Y-QH helped to revise the manuscript.

## Conflict of Interest Statement

The authors declare that the research was conducted in the absence of any commercial or financial relationships that could be construed as a potential conflict of interest.

## Footnotes

1 http://www.bioinformatics.babraham.ac.uk/projects/fastqc/

2 https://sourceforge.net/projects/bbmap/

3 https://bitbucket.org/berkeleylab/metabat

4 https://github.com/Ecogenomics/CheckM

5 http://broadinstitute.github.io/picard/
