# Supplementary material for: Metagenomic Evidence for a Methylocystis Species Capable of Bioremediation of Diverse Heavy Metals
Source: Front Microbiol. 2019 Jan 9;9:3297. doi: 10.3389/fmicb.2018.03297 (PMC6333641; doi:10.3389/fmicb.2018.03297)
Supplement: Supplementary file 1 [file Data_Sheet_1.pdf]

# Metagenomic evidence for a *Methylocystis* species capable of bioremediation of diverse heavy metals

Ling-Dong Shi[1, 2, 3], Yu-Shi Chen[1], Jia-Jie Du[1], Yi-Qing Hu[1], James P. Shapleigh[4], He-Ping Zhao[1, 2, 3,]*

1. College of Environmental and Resource Science, Zhejiang University, Hangzhou, China.
2. Zhejiang Prov Key Lab Water Pollut Control & Envi, Zhejiang University, Hangzhou, Zhejiang, China.
3. MOE Key Lab of Environmental Remediation and Ecosystem Health, College of Environmental and Resource Science, Zhejiang University, Hangzhou, China, 310058.
4. Department of Microbiology, Cornell University, Ithaca, NY, United States.

* Correspondence to Dr. He-Ping Zhao. Tel (Fax): 0086-571-88982739, E-mail: zhaohp@zju.edu.cn

**SI Methods:**

10 mL of the consortium containing high abundance of *Methylocystis* sp. HL18 as we reported in the manuscript was added into the 50-mL serum bottle with 25 mL of the fresh mineral medium consisted of ~100 μM As(V). After degassing with the argon, 10 mL of methane was injected into the sealed bottles. We took ~0.4 mL liquid samples every day using 1-mL syringe and filtrated using a 0.22 μm membrane filter (LC+PVDF membrane, Shanghai Xinya, China) immediately. As(V) was measured through ion chromatography (Dionex ICS-1000, ThermoFisher Scientific) with an AS 19 column and AG 19 guard column. The system parameters were set as 20 mM KOH of eluent concentration and 1 mL/min of flow rate. Meanwhile, similar experiments except for no methane addition or with killed inoculum were also constructed as negative controls.

**Table S1.** Classification and abundance of recovered provisional whole genome sequences with abundance >5.0%. The sum of them accounted for >70% of the community.

| **Classification** | **Abundance (%)** |
|---|---|
| *Pseudoxanthomonas* sp. | 18.9 |
| *Meiothermus* sp. | 17.5 |
| *Thermomonas* sp. | 12.8 |
| ***Methylocystis* sp. HL18** | 9.9 |
| *Aquimonas* sp. | 7.4 |
| *Methylophilus* sp. | 6.6 |
| Sum | 73.1 |

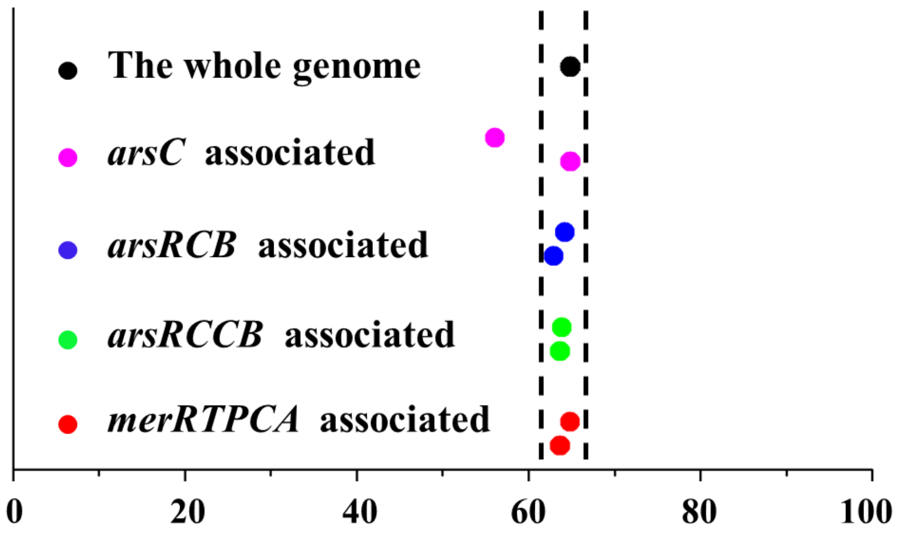


**Figure S1.** GC contents of individual operons, contigs and the whole genome. Within every type (color), the upper one and bottom one indicated values of the functional gene operon and the relevant contig, respectively.

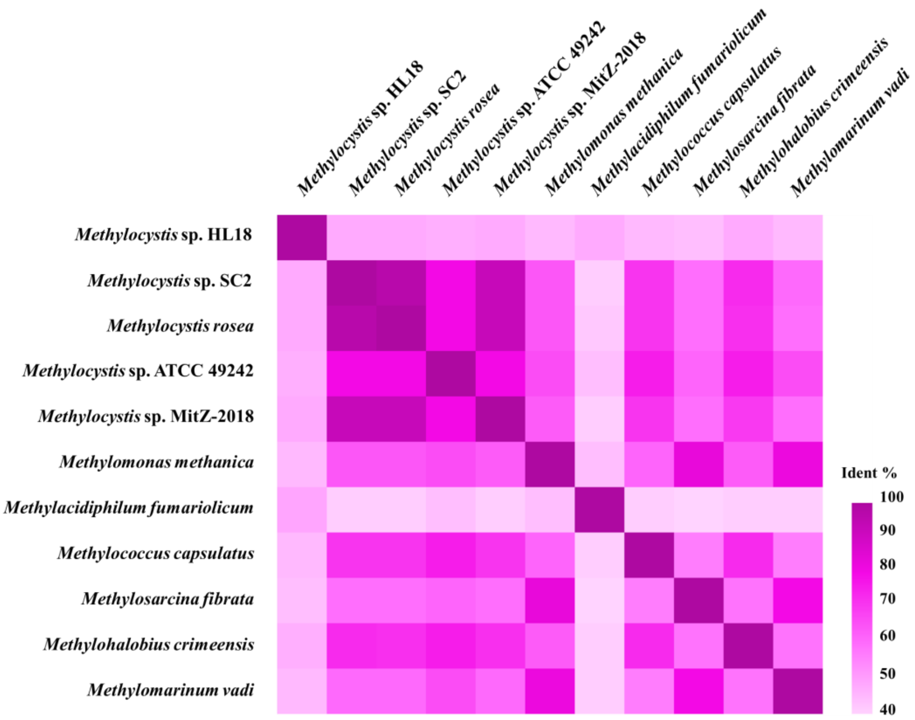


**Figure S2.** Heatmap of MerA identities from representative species of different methanotrophic genera.

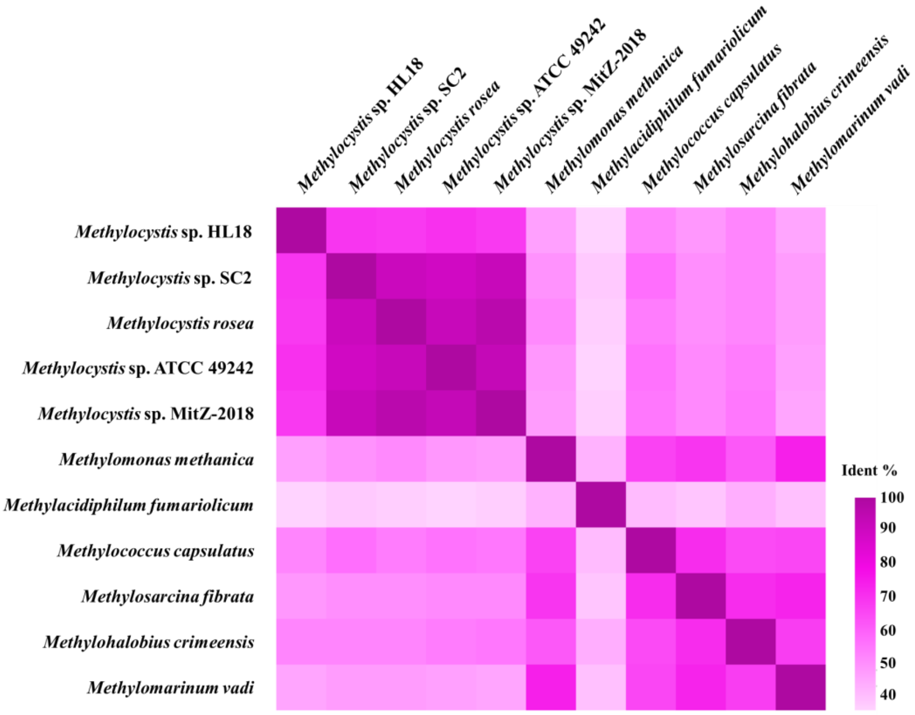


**Figure S3.** Heatmap of glutaredoxin-clade ArsC identities from representative species of different methanotrophic genera.

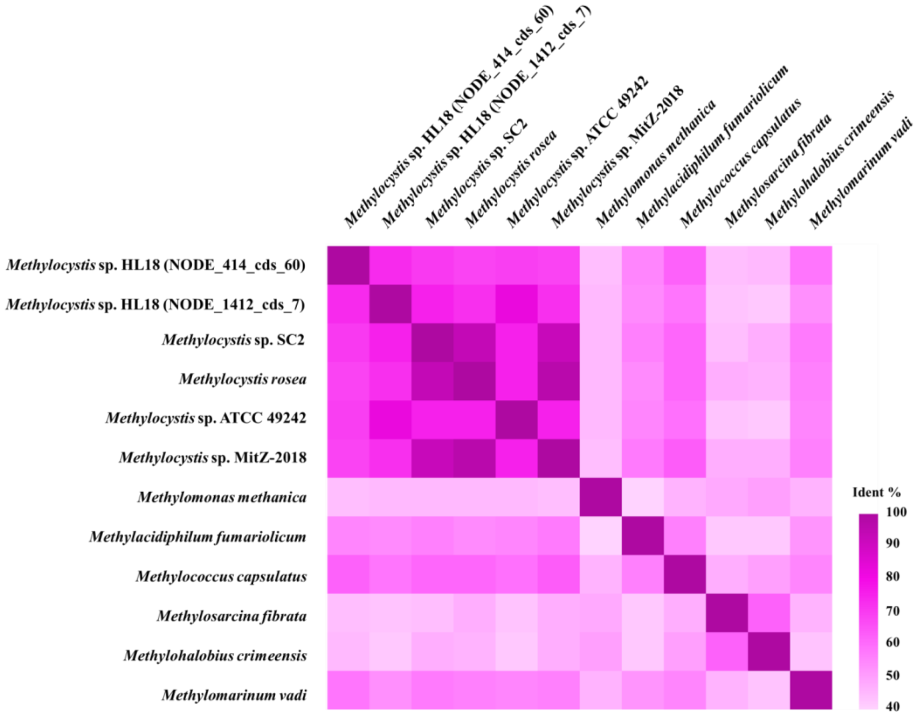


**Figure S4.** Heatmap of thioredoxin-clade ArsC identities from representative species of different methanotrophic genera.

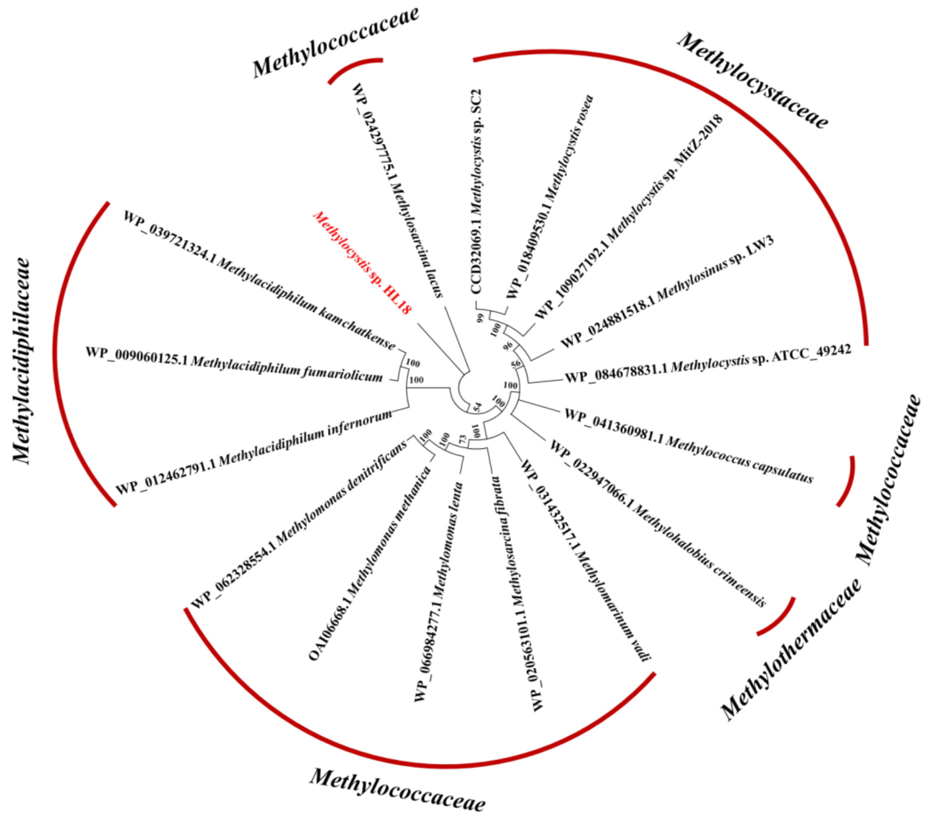


**Figure S5.** Phylogenetic tree of MerA based on amino acid sequences from different methanotrophic genera. Numbers indicated the bootstrap support (1000 replicates), which were filtered when less than 50%.

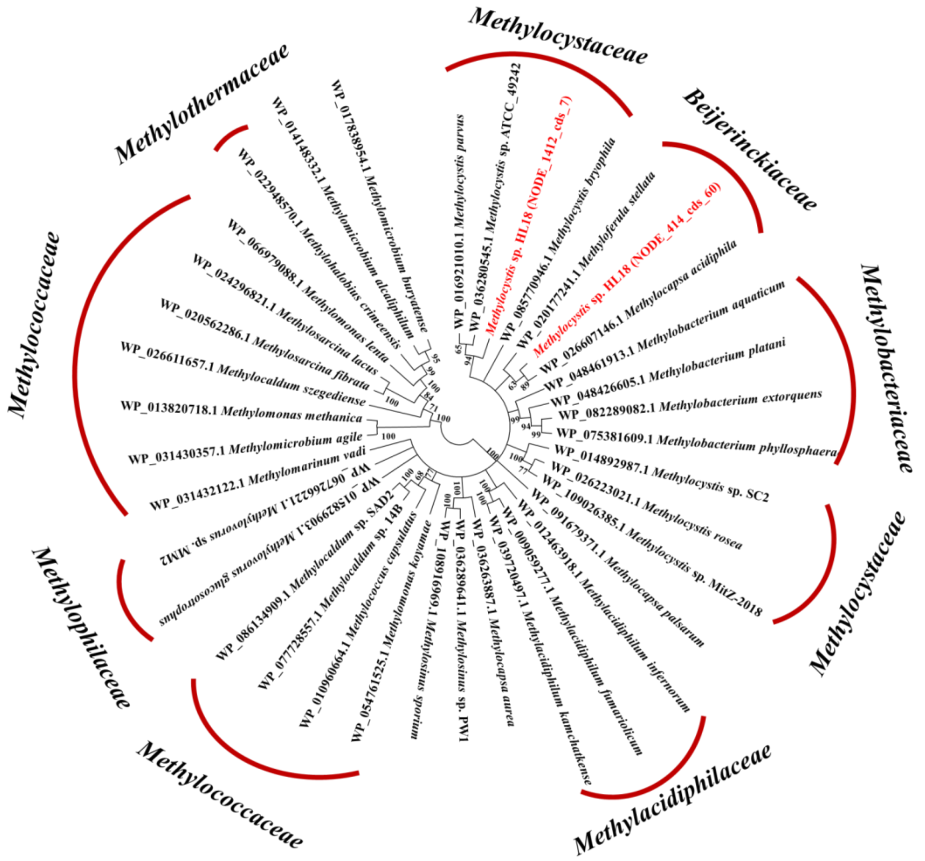


**Figure S6.** Phylogenetic tree of thioredoxin-clade ArsC based on amino acid sequences from different methanotrophic genera. Numbers indicated the bootstrap support (1000 replicates), which were filtered when less than 50%.

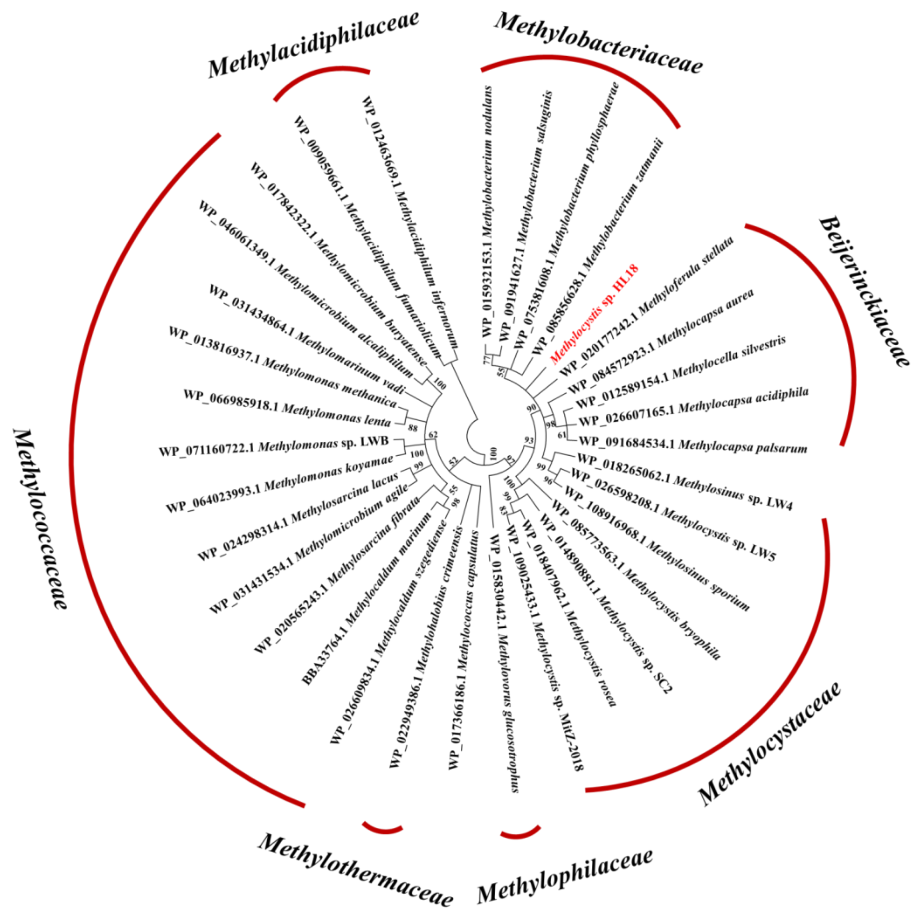


**Figure S7.** Phylogenetic tree of glutaredoxin-clade ArsC based on amino acid sequences from different methanotrophic genera. Numbers indicated the bootstrap support (1000 replicates), which were filtered when less than 50%.

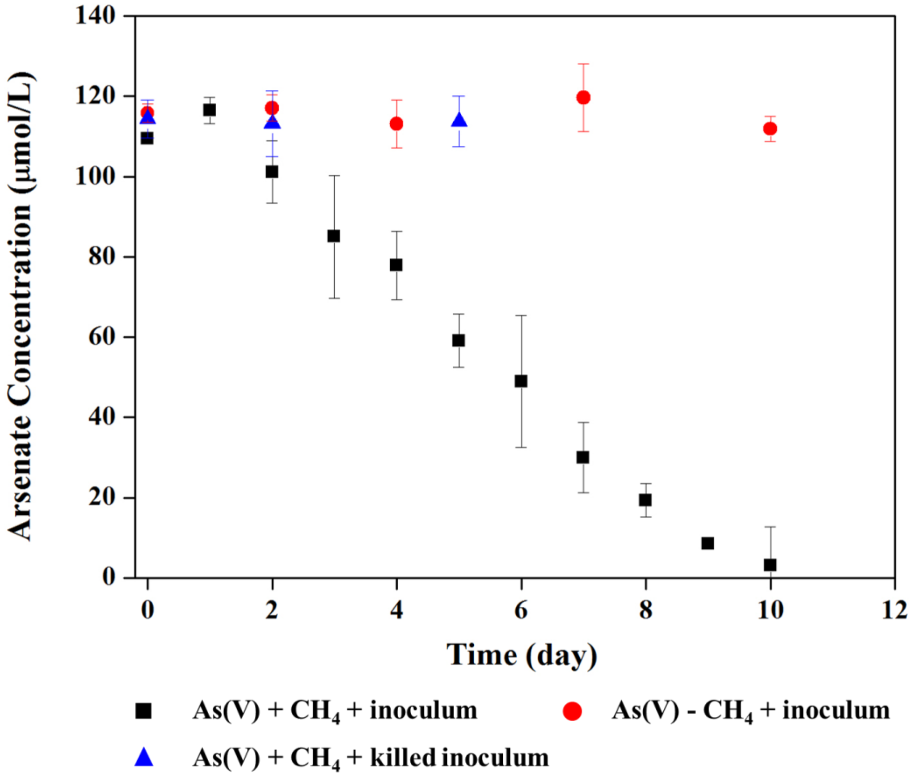


**Figure S8.** Arsenate reduction by HL18-containing consortium tested in serum bottles under different conditions. Error bar indicated the mean deviation of duplicate.
